# miR-34 regulates cuticle pigmentation by targeting *Bm-iAANAT* and *Bmserpin3* in *Bombyx mori*

**DOI:** 10.1080/15476286.2026.2675852

**Published:** 2026-05-26

**Authors:** Zulian Liu, Dehong Yang, Xingyu Luo, Yongping Huang

**Affiliations:** School of Environmental Science and Engineering, Shanghai Jiao Tong University, Shanghai, China

**Keywords:** Silkworm, miR-34, pigmentation, tyrosine metabolism, melanin

## Abstract

Pigmentation is an orchestrated process involved in cuticle melanization and sclerotization in insects, and plays a critical role in maintaining the structural integrity and functional completeness of the insect cuticle. Although the cascade reactions underlying pigmentation have been extensively studied, our understanding of the involvement of miRNA in this process remains limited. Here, we investigate the role of conserved microRNA-34 (miR-34) in regulating cuticular colouration in the silkworm, *bombyx mori*. Overexpression of miR-34 led to pronounced melanization in the larval abdomen. Mechanistically, LC/MS analysis revealed that miR-34 overexpression alters the epidermal amino acid composition, with a particularly notable increase in tyrosine and dopamine content. Enzyme activity assays confirmed the activation of phenoloxidase (PO). Through experimental validation, we identified two key target genes of miR-34, including *Bm-iAANAT* (a critical gene in melanin synthesis) and *Bmserpin3* (a regulator of the serine protease cascade). This study uncovers a previously unreported miRNA mediated regulatory mechanism in insects and systematically elucidates how miR-34 coordinately modulates pigmentation through distinct molecular pathways, providing novel theoretical insights into this field.

## Introduction

Insects play vital roles in ecosystem functioning due to their remarkable environmental adaptability, sophisticated communication systems, and evolutionarily optimized body structures and functions. Among these traits, colouration stands out as one of the most variable characteristics in insects. Pigmentation, in particular, contributes to critical adaptive processes such as mimicry, sexual selection, thermoregulation, preventing ultraviolet damage, and other ecological interactions across diverse insect groups [1].

Lepidoptera, one of the most species rich orders within the class Insecta, comprises two major evolutionary lineages, moths and butterflies, and ranks as the second most widely distributed insect group globally, surpassed only by Coleoptera. Approximately most of lepidopteran species hold significant agricultural and economic importance, including numerous devastating pests. Their chromatic polymorphism stands as a paradigm in the animal kingdom, with this phenotypic diversity conferring distinct evolutionary advantages in species recognition, environmental adaptation, and survival strategies. Deciphering the molecular basis and regulatory networks underlying their pigmentation not only advances fundamental understanding of insect developmental biology but also identifies potential targets for pest control. Research demonstrates that larval colouration undergoes dynamic changes tightly coupled with moulting cycles, each ecdysis event triggers metabolic reprogramming of pigments under precise regulation by the 20-hydroxyecdysone (20E) hormone signalling pathway [2,3]. Furthermore, studies reveal that the intricate colour phenotypes in Lepidoptera arise from cascading interactions of multiple pigment systems. Among these, the melanin biosynthesis pathway, mediated by melanosome organelles, exhibits remarkable evolutionary conservation [2,3].

The melanization process often coincides with cuticular sclerotization, with pathological melanism generally manifesting as abnormal ectopic deposition of melanin pigments [4]. The melanization process involves several key enzymatic steps: *Tyrosine hydroxylase* (*TH*), the rate-limiting enzyme in catecholamine biosynthesis, catalyzes the hydroxylation of tyrosine to L-DOPA, representing the initial step in both pigmentation and sclerotization. *Dopa decarboxylase* (*DDC*) converts L-DOPA to dopamine, a critical precursor for both cuticle hardening and melanin formation. *Arylalkylamine N-acetyltransferase* (*AANAT*) mediates the conversion of dopamine to N-acetyldopamine, a key intermediate in melanin synthesis [5,6]. In most invertebrates, melanin synthesis occurs through the phenoloxidase (PO)-activating system, which plays essential roles in innate immunity, sclerotization and pigmentation. This biochemical cascade generated toxic quinone intermediates and o-quinones that can potentially harm the hosts organism. Consequently, PO-mediated melanin synthesis requires precise regulation to prevent pleiotropic effects on normal development and pigmentation patterns.

MicroRNAs (miRNAs) are a class of small RNAs (18–25 nucleotides) that are evolutionarily conserved across animals, plants, insects [7]. Despite their small size, miRNAs play pivotal regulatory roles in diverse biological processes such as apoptosis [8], cell cycle progression [9], Notch signalling [10], neural development [11] and oogenesis [12]. Notably, our previous work demonstrated that the highly conserved miR-34 regulates larval growth and wing morphogenesis in *Bombyx mori* by directly modulating ecdysone signalling and cuticle protein expression [13]. Here, we reveal a novel role for miR-34 in cuticular pigmentation and sclerotization progress. The miR-34 overexpression (miR-34-OV) strains exhibited distinct pigment-related phenotypes including epidermal darkening, cuticle thinning, elevated tyrosine and dopamine levels, and increased PO activity. To investigate miR-34^’^s role in the pigmentation, we conducted RNA-seq analysis comparing the miR-34-OV and wild-type (WT) silkworms, identifying differentially expressed genes associated with melanin biosynthetic process. Through a combination of luciferase reporter assays and qPCR validation, we identified *Bm-iAANAT* and *Bmserpin3* as direct targets of miR-34, which involved in the melanin biosynthetic and serine protease cascades, respectively. This finding provided new mechanistic insight into the regulation of insect cuticular pigmentation and sclerotization.

## Results

### Ubiquitous ectopic expression of miR-34 triggers abdominal melanization and cuticle thinning in larval development

**Figure 1.**
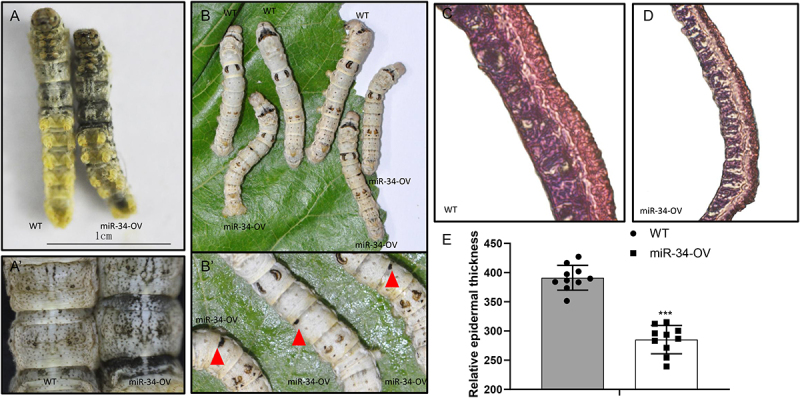
Ectopic expression of miR-34 results in increases melanin deposition and thin cuticle. (A) and (A’). The abdomen of L4D4 larvae excessive for miR-34 develop a spontaneous melanized reaction. (B) and (B’). The melanized nodules appeared in miR-34-OV silkworms. (C and D) the cuticle thickness of the larval in miR-34-OV decreased compared to the wild-type. (E) Comparison of epidermal thickness between miR-34-OV and WT silkworms (*n* = 10 data sets).

To explore the functional role of miR-34 in vivo, we conducted genetic analysis and observed that ubiquitous overexpression of miR-34 in transgenic silkworm lines (miR-34-OV) induced distinct body colour modifications. Compared to WT controls, miR-34-OV larvae exhibited pronounced melanization, particularly in the abdominal segments (Figures 1(A, A’)). Additionally, the miR-34-OV group enhanced integument spot formation (Figure 1(B)). These phenotypic changes were consistently detectable from the early third-instar to late fifth-instar larval stages. Notably, miR-34-overexpressing larvae developed melanized nodules at the segmental epidermal boundaries (Figures 1(B, B’)), a feature absent in WT specimens.

Sclerotization in insects, a biochemical process closely associated with yet distinct from melanization, proceeds concurrently through independent pathways. This dual process is vital for insect survival, as larval colouration patterns are fundamentally governed by the biochemical composition and spatial distribution of pigments within the procuticle and exocuticle layers [14]. To investigate whether cuticle sclerotization is associated with differences in miR-34-OV expression and WT silkworms, we analysed the ultrastructure of the epidermis in fifth instar larvae after ecdysis. Our results showed that miR-34-OV larvae exhibited reduced cuticle thickness compared to WT silkworms (Figures 1(C-E)).

Given that ubiquitous ectopic expression of miR-34 triggers abdominal melanization in the silkworm, we further investigated whether miR-34 exhibits differential expression between the black and white regions of the epidermis. However, the results showed that the expression levels of miR-34 in these two regions were not significantly different (Supplement Figure 1(A-B)).

### Ectopic expression of miR-34 affects the regulation of tyrosine metabolism in silkworms

**Figure 2.**
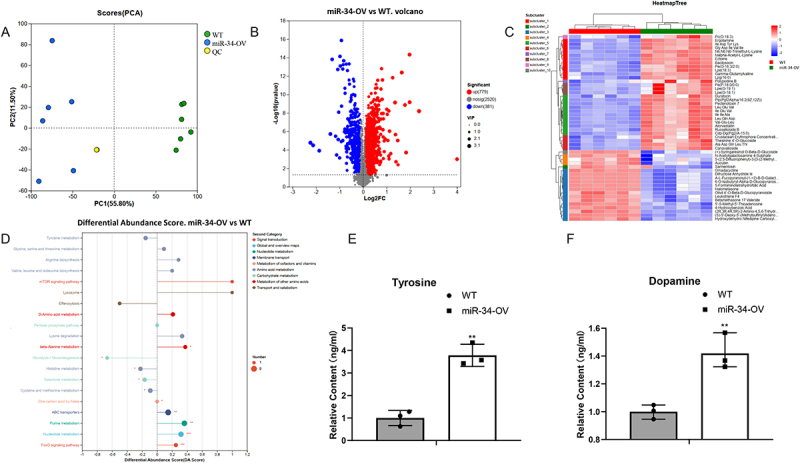
Ectopic expression of miR-34 affects the regulation of tyrosine metabolism in silkworms. (A) and (B) PCA and volcano plots revealed distinct metabolite profiles between miR-34-OV and WT silkworms. (C) Hierarchical clustering of differential metabolites across different samples. (D) KEGG enrichment analysis of differential metabolites associated with melanin deposition in silkworm. (E) and (F) Relative content of tyrosine and dopamine in miR-34-OV and wt silkworm larval. The asterisks ** indicate significant differences (*p* < 0.01) compared with the wt. The data were obtained from three independent experiments.

Given the observed abdominal melanization and cuticle thinning during larval development, we performed untargeted liquid chromatography-mass spectrometry (LC-MS) metabolomic analysis of cuticle samples from both miR-34-OV and WT silkworms to identify endogenous metabolites mediating melanization and sclerotization. Principal component analysis (PCA) and volcano plots revealed distinct metabolite profiles between miR-34-OV and WT silkworms (Figure 2(A, B)). Metabolomic profiling identified alterations in 1,160 metabolites, with 779 upregulated and 381 downregulated in the miR-34-OV group. Hierarchical clustering analysis of significantly differential metabolites demonstrated a predominant association with amino acid metabolism (Figure 2(C)). Notably, KEGG pathway enrichment analysis highlighted the tyrosine metabolism pathway as particularly significant (Figure 2(D)), given its well established role in insect melanization and related physiological regulation.

To further investigate tyrosine metabolism dysregulation, we quantified tyrosine and dopamine, key pathway metabolites, using LC-MS. The analysis showed significantly elevated tyrosine and dopamine levels in the cuticular layer of miR-34-OV silkworms compared to WT controls (Figure 2(E, F)). Importantly, these metabolic differences correlated with the observed phenotypic divergence in abdominal melanization and cuticle thickness between the two groups.

### miR-34 affects the expression of melanin biosynthetic cascade genes

**Figure 3.**
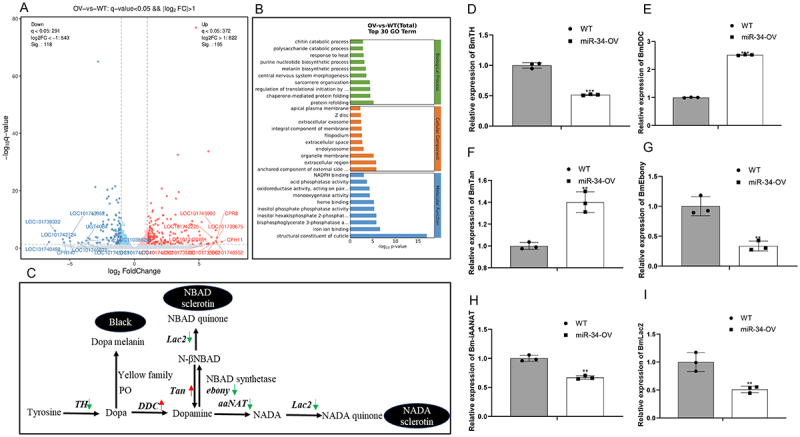
miR-34 affects the expression of melanin biosynthetic cascade genes. (A) Volcano plots of differentially expressed genes of miR-34-OV and WT silkworm.The red and blue dots denote upregulated and downregulated genes, respectively. (B) GO analysis of differentially expressed genes between miR-34-OV and WT revealed the top 30 enriched pathways. (C) The canonical melanin biosynthetic cascade, the red arrows were represented the upregulated, and the green arrows were represented the downregulated. The six genes, (D) *BmTH*, (E) *BmDDC*, (F) *BmTan*, (G) *Bmebony*, (H) *BmiAANAT*, (I) *BmLac2*, were detected in the miR-34-OV and wt silkworms by qPCR. The asterisks ** and *** indicate significant differences (*p* < 0.01 and *p* < 0.001, respectively) compared with the WT. The data were obtained from three independent experiments.

To investigate the molecular mechanisms underlying abdominal melanization and cuticle thinning during larval development, we conducted comparative transcriptomic analysis of epidermis tissues from miR-34-OV and WT silkworms. RNA sequencing revealed 384 differentially expressed genes (DEGs) meeting our significance threshold (FDR q < 0.05), comprising 145 down-regulated and 239 up-regulated genes in the miR-34-OV silkworms (Fig, 3A). GO pathway analysis identified two significantly enriched biological processes, chitin catabolic process, melanin biosynthetic pathway, showing marked differences between groups (Figure 3(B)). The melanin biosynthetic pathway, a well-characterized regulator of insect pigmentation (Figure 3(C)), exhibited particularly pronounced changes. We focused on six key synthesis genes, significant downregulation of *BmTH* (Figure 3(D)), *Bmebony* (Figure 3(G)), *Bm-iAANAT* (Figure 3(H)), and *Bmlac2* Figure 3I) in miR-34-OV silkworms. Conversely, *BmDDC*, the crucial enzyme for cuticular pigmentation and sclerotization, showed significant upregulation (Figure 3(E)). The gene *BmTan*, was also significantly upregulated which involved in cuticle tanning in epidermis (Figure 3(F)).

Although the exact biological function of the yellow family genes remains incompletely characterized, previous studies have established its essential role in cuticular melanin deposition [15,16]. Our analysis revealed significant alterations in the expression of yellow family genes (Supplement Figure 2(A-H)). Together, these finding suggest that miR-34 serves as a key regulator of the melanin biosynthetic pathway.

### Activation and regulation of the PO system by miR-34

Melanization is a critical biochemical process in insects that orchestrates diverse physiological functions, notably including cuticular sclerotization. This process is initiated when microbial pattern recognition receptors detect external challenges, subsequently activating a cascade of serine protease reactions. These sequential enzymatic activations culminate in the proteolytic conversion of prophenoloxidase (PPO) to its active form, phenoloxidase (PO), which serves as the central regulatory enzyme controlling melanin biosynthesis [17,18].

**Figure 4.**
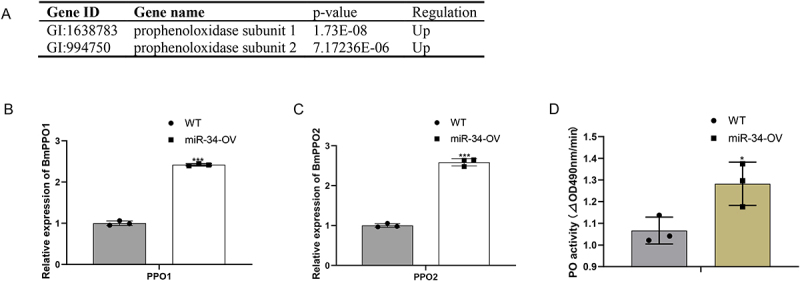
Activation and regulation of the PO system by miR-34. (A) Expression of *BmPPO1* and *BmPPO2* in miR-34-OV silkworm were detected by RNA-seq. (B) The expression of *BmPPO1* and *BmPPO2* were detected by qPCR. (C) More than five silkworms larval for the sample per group were used for measurements of PO activity. The asterisks * and *** indicate significant differences (*p* < 0.05 and *p* < 0.001, respectively) compared with the WT. The data were obtained from three independent experiments.

In *Bombyx mori*, the PPO system involves two key genes: *BmPPO1* and *BmPPO2* [19]. To investigate the molecular basis of the observed pigmentation phenotype in miR-34-OV larvae, we examined miR-34’s regulatory effects on PPO expression. Our analysis revealed significant upregulation of both *BmPPO1* and *BmPPO2* following miR-34-OV larvae compared to WT controls (Figure 4(A-C)). Consistent with these transcriptional changes, we observed a substantial increase in PO enzymatic activity in miR-34-OV larvae (Figure 4(D)), which correlated directly with the elevated PPO transcript levels. These results establish that miR-34 activated the PO system in silkworm larvae by transcriptionally upregulating *BmPPO1* and *BmPPO2*, thereby contributing to melanin biosynthesis and modulating cuticular pigmentation.

### miR-34 targets components of the melanin biosynthesis and serine protease signalling pathways

**Figure 5.**
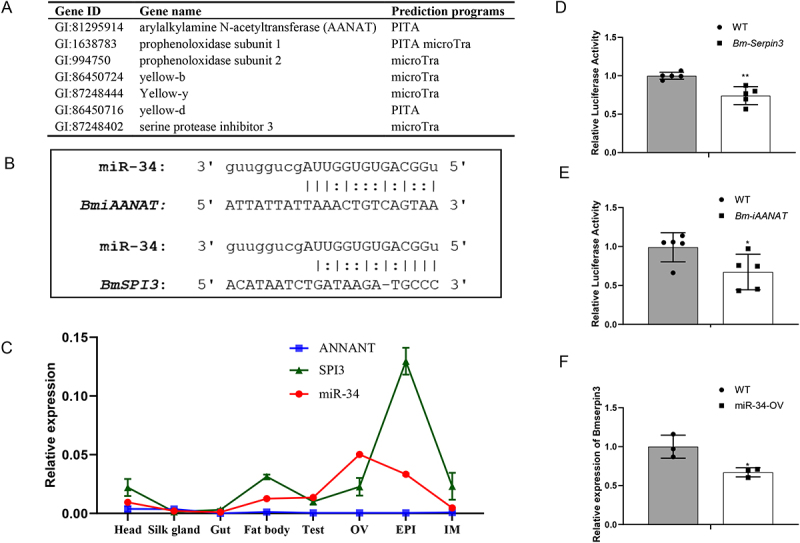
In vitro and vivo validations of miR-34 target genes. (A) The putative target genes in melanin biosynthetic cascade. (B) Potential miR-34 binding sites within the 3’UTRs of the candidate target genes *Bm-iAANAT* and *Bmserpin3*. (C) The relative expression of miR-34 and its target genes, *BmiAANAT* and *Bmserpin3*, as detected by qPCR, exhibited opposite trends across various tissues. (D) and (E) miR-34 significantly reduced the relative luciferase activity of reporter constructs containing the 3’-UTRs of *Bm-iAANAT* and *Bmserpin3*. (F) The expression of *Bmserpin3* in miR-34-OV and WT silkworms was detected by qPCR. The asterisks * and ** indicate significant differences (*p* < 0.05 and *p* < 0.01, respectively) compared with the WT. The data were obtained from at least three independent experiments.

MicroRNAs mediate gene expression post-transcriptionally through binding to the untranslated regions (UTRs) of target mRNAs, primarily via their seed region [20]. In the silkworms, miR-34 overexpression disrupted the transcriptional regulation of genes involved in the melanin biosynthetic pathway (Figure 3). To identify potential targets of miR-34 within this pathway, we performed computational prediction using two independent algorithms, PITA and microTra (Figure 5(A)). We found that miR-34 directly targets the *Bm-iAANAT* and *Bmserpin3* (Figure 5(B)). Subsequently, we analysed the tissue-specific expression profiles of miR-34 and its target genes *Bm-iAANAT* and *Bmserpin3* in the silkworm. The results revealed an inverse regulatory relationship between miR-34 and its target genes *Bm-iAANAT* and *Bmserpin3*, which is consistent with the canonical miRNA regulatory pattern (Figure 5(C)).

The putative target genes were further validated using a dual luciferase reporter assay in HEK293 cells. miR-34 significantly reduced the relative luciferase activity of reporter constructs containing the WT 3’-UTRs of *Bm-iAANAT* and *Bmserpin3*, whereas no effect was observed with a control plasmid (Figure 5(D, E)). These results demonstrate that *Bm-iAANAT* and *Bmserpin3* are direct targets of miR-34. To corroborate these findings, we analysed *Bm-iAANAT* and *Bmserpin3* expression in miR-34-OV silkworms. Consistent with the reporter assay results, the qPCR analyses provide compelling additional evidence that miR-34 significant downregulated of both genes at the mRNA level in miR-34-OV silkworms (Figures 3(H), 5(F)). Together, these data confirm that *Bm-iAANAT* and *Bmserpin3* are directly regulated by miR-34 in silkworm.

## Discussion

**Figure 6.**
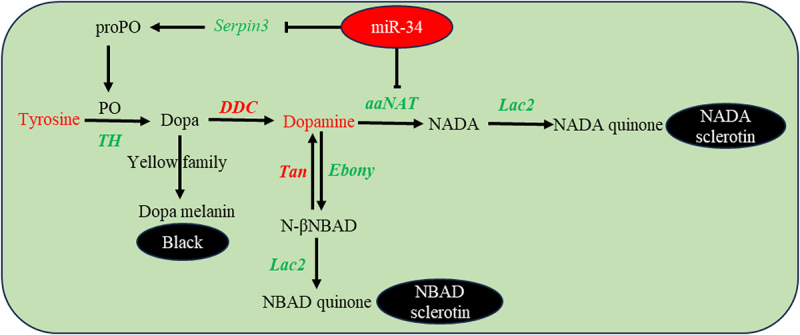
Schematic diagram of miR-34-mediated regulation in the melanization pathway and PO activation system. In the graphical representation, the following colour scheme was employed: amino acids are depicted in blue, while upregulated and downregulated gene identifiers are highlighted in red and green, respectively.

This study demonstrates that miR-34 plays a role in cuticular melanization and sclerotization in *Bombyx mori*. Specifically, miR-34 targets and suppresses *Bm-iAANAT*, a key gene in melanin biosynthesis, thereby modulating the melanization pathway. Concurrently, it affects serine protease cascades by targeting the serine protease inhibitor *Bmserpin3* (Figure 6). This coordinated regulatory mechanism provides novel insights into the molecular network governing cuticular pigmentation in insects.

The enzyme *AANAT* plays a crucial role in melanin synthesis by catalysing the conversion of dopamine to N-acetyldopamine in insects [21]. In the silkworm, two distinct *AANAT* isoforms have been characterized. Genetic studies have demonstrated that loss-of-function mutants (*Bm-iAANAT*), result in dopamine accumulation, producing the characteristic melanic phenotype observed in the melanism (*mln*) mutant [22,23]. Similarly, knocking down of the *Bm-iAANAT2* alters dopamine homoeostasis and induces ectopic black pigmentation in the head capsule [24]. miR-34-overexpressing silkworms exhibit increased melanin deposition in the epidermis. Target gene prediction and experimental validation revealed that miR-34 targets and regulates *Bm-iAANAT*, leading to downregulation of its transcript level. These findings provide direct molecular evidence that miR-34 mediates the melanization pathway via *Bm-iAANAT*.

Serine protease inhibitors (serpins) represent an evolutionarily conserved protein superfamily that regulates critical physiological processes. In silkworm, multiple serpin genes have been molecularly characterized, with several members functionally elucidated [25,26]. Notably, the silk gland-specific serpins *Bmserpin-16* and *Bmserpin-18* exhibit potent inhibitory activity against cysteine proteinase, suggesting their potential role in modulating fibroinase activity during silk production [27]. Genetic evidence implicates *Bmserpin-2* in antiviral defence mechanisms [28], while *Bmserpin-15* has been shown to negative regulation of both PPO activation and antimicrobial peptides (AMPs) synthesis [29]. Beyond their established roles in immunity, serins critically govern melanization cascades and AMP production. Previous studies have demonstrated that the serine protease inhibitor *Bmserpin3* serves as a crucial negative regulator of prophenoloxidase (PPO) activation during larval development [30]. This study reveals for the first time that miR-34 suppresses *Bmserpin3* expression at the transcriptional level, leading to a significant enhancement of PPO enzymatic activity. Notably, the direct inhibitory effect of *Bmserpin3* on PPO activity shows a high degree of consistency with previous research findings.

In the miR-34-OV silkworms, the directly suppressed target genes *Bm-iAANAT* and *Bmserpin3* are downregulated, leading to dysregulation of the melanin synthesis pathway. We supposed that this dysregulation results in a blockage at either the upstream or middle stages of the pathway, thereby causing abnormal accumulation of melanin precursors such as tyrosine and dopamine. Since high concentrations of these precursors are cytotoxic and induce oxidative stress [31,32], cells may respond by feedback regulating the expression network of melanin synthesis related genes through an unknown sensing pathway, aiming to consume excess precursors or redirect metabolic flux. This suggests that the expression changes of most melanin synthesis genes are secondary responses to metabolic imbalance. In contrast, upon knockout of miR-34 using the CRISPR/Cas9 transgenic system, no obvious changes in body colour or epidermal thickness were observed in the transgenic silkworms [13]. We speculate that other functionally redundant microRNAs (e.g. overlapping non-coding RNAs) in the silkworm genome can compensate for the loss of miR-34 to sustain normal expression of melanin synthesis related genes. However, upon overexpression of miR-34 beyond physiological levels, this redundancy is insufficient to compensate, resulting in an observable phenotype. This observation is not contradictory. We propose that miR-34 function does not depend on its basal expression difference between the two regions, but instead on the state and plasticity of its downstream target gene network.

At the same time, miR-34 has been previously characterized as a regulator of larval growth and wing morphogenesis through its modulating ecdysone signalling and cuticle protein expression in *Bombyx mori* [13]. Our study uncovers that it modulates the melanin pathway, consequently affecting melanin deposition in *Bombyx mori*. These multifunctional properties correlate well with endocrine mechanisms controlling insect cuticular pigmentation. In summary, our comprehensive molecular analyses establish miR-34 as a key regulatory element within the intricate network governing melanization in *Bombyx mori*.

## Methods and materials

### Silkworm rearing

The miR-34 ubiquitous overexpression transgenic strain (miR-34-OV) has been previously described [13]. In brief, transgenic silkworms were generated using *piggyBac*-based transgenic plasmids. The final transgenic construct, *pBac* [*IE1*-EGFP-*IE1*-miR-34], contained the miR-34 sequence comprising Bm-pre-miR-34 and the 5’- and 3’-end flanking sequences from genomic DNA.

To obtain transgenic silkworms, transformation plasmids together with the *piggyBac* helper plasmid were microinjected into pre-blastoderm G_0_ embryos using standard procedures. The injected embryos were incubated at 25°C in a humidified chamber for 10–12 days until hatching. The hatched larvae were reared to adulthood and then either crossed with WT moths or sib-mated. G_1_ (transgenic strain) progeny were screened for the presence of the marker gene during the embryonic stage under a fluorescence microscope. miR-34-OV and WT silkworms were reared on fresh mulberry leaves at 25°C and 75% relative humidity under a 12-h dark and 12-h light photoperiod.

### Transmission election microscopy (TEM)

The epidermis was dissected from freshly collected day-3 instar larvae and fixed overnight at 4% in Qurnah’s fixative (ethanol: chloroform: acetic acid = 6:3:1, v/v). Following fixation, samples were dehydrated through a graded ethanol series. Materials were then embedded and sectioned to slices (five slices/sample) using a Leica Section Machine (RM2235). Sections were dewaxed in xylene and rehydrated sequentially through 95%, 80%, and 70% ethanol solutions, followed by distilled water and phosphate-buffered saline (PBS). Haematoxylin and eosin staining (HE) (Beyotime; 10 mg/mL) staining was performed under an Olympus BX51 microscope.

### RNA-sequencing (RNA-seq) analysis

Total RNA was extracted from the epidermal tissue of individual animals on the second day of the fifth instar in both miR-34-OV and WT groups. Three biological replicates were sequenced for each group. For mRNA sequencing, total RNA was enriched and fragmented prior to cDNA synthesis and library preparation. The resulting libraries were sequenced on the Illumina HiSeq 2000 platform. Raw sequencing data underwent quality control, filtering, alignment, and quantification using FastQC, Trimmomatic, Bowtie2, and RSEM, respectively, with alignment to the *Bombyx mori* reference genome (available at https://silkdb.bioinfotoolkits.net).

To identify DEGs between the two groups, transcript expression levels were calculated using the transcripts per million (TPM) method, and gene abundance was quantified with RSEM [33]. Additionally, functional enrichment analyses, including Gene Ontology (GO) and KEGG pathway analyses, were performed to determine significantly enriched GO terms and metabolic pathways among DEGs (Bonferroni-corrected *p* < 0.05).

All RNA-seq data have been deposited in the NCBI Sequence Read Archive (SRA) under BioProject accession number PRJNA1453063. The raw sequencing data for all six samples are available under accession numbers SRR38085335 and SRR38085340.

### Quantitative real-time PCR (qRT-PCR)

Total RNA was extracted from silkworm and tissue samples using TRIzol (YESEN, China) according to the instruction manual, the quantity of purified RNA was detected micro-spectrophotometer (Nanodrop 300, China), and integrity was analysed by agarose gel. cDNA synthesis using Hiscript II Reverse Transcriptase kit (Vazyme, China) for qPCR (ABI StepOnePlus Real-Time PCR System, Singapore). qPCR was performed using SYBR Green Supermix. All analyses were normalized based on amplification of silkworm *BmRp49*. Primer sequences for qPCR are listed in Table S1.

### LC-MS

Tyrosine (T2900000) and Dopamine (H8502-5 G), were purchased from Sigma-Aldrich. Quantitative analysis of these characteristic chemical components was identified by LC-MS using a Q Exactive Plus system (small molecule mode) with three biological replicates per group. For sample preparation, fourth instar larvae during apolysis were homogenized in the extraction solution (water: acetonitrile = 1:1 (v:v)) and incubated at 4°C for 24 h. The homogenates were then centrifuged at 13,000 g at 4°C for 15 min, and the resulting supernatant was subjected to LC-MS analysis.

### Phenoloxidase activity assay

Haemolymph samples were collected from six day-2 fifth-instar larvae (L5D2) and dispensed into 1.5 mL microcentrifuge tube (Axygen), After centrifugation at 500 ×g for 10 min in a 4°C, the supernatant was carefully collected. For enzymatic activity assays, 1.5 μL of haemolymph was mixed with 200 μL of L-dopamine (10 mmol/L) in a 96-well plate. Absorbance was immediately measured at 490 nm using a micro-spectrophotometer (Nanodrop 300, China). Three independent biological replicates were evaluated for each experimental group.

### miRNA target computational prediction and reporter plasmid construction

Putative miRNAs targets were predicted using two computational algorithms, PITA and microTra, in combination, thresholds were set as follows: a ddG ≤0 for PITA, and an energy ≥0.5 for microTar, following previously described protocols [34].

For the luciferase reporter assay, 293T cells were grown to 80% confluence and co-transfected with the reporter vector mixture. Relative luciferase activity was measured using a dual luciferase reporter assay kit (Promega, USA, # E1910).

### Statistical analysis

GraphPad Prism 8 was used for data analysis and graph generation. The *t*-test was used to determine significant differences. *p*-values were categorized as follows: *p < 0.05; **p < 0.01; ***p < 0.001, not significant was represented as ns. All data were obtained from at least three independent experiments.

## Supplementary Material

Supplemental Material

## Data Availability

The authors confirm that the data supporting the findings of this study are available in the article and its supplementary materials. Any further underlying data will be made available upon reasonable request.

## References

[1] True JR. Insect melanism: the molecules matter. Trends Ecol Evol. 2003;18(12):640–647. doi: 10.1016/j.tree.2003.09.006

[2] Futahashi R, Fujiwara H. Regulation of 20-hydroxyecdysone on the larval pigmentation and the expression of melanin synthesis enzymes and yellow gene of the swallowtail butterfly, *Papilio xuthus*. Insect Biochem Mol Biol. 2007;37(8):855–864. doi: 10.1016/j.ibmb.2007.02.014 17628284

[3] Hiruma K, Riddiford LM. The molecular mechanisms of cuticular melanization: the ecdysone cascade leading to dopa decarboxylase expression in *Manduca sexta*. Insect Biochem Mol Biol. 2009;39(4):245–253. doi: 10.1016/j.ibmb.2009.01.008 19552890

[4] Whitten MMA, Coates CJ. Re-evaluation of insect melanogenesis research: views from the dark side. Pigment Cell Melanoma Res. 2017;30(4):386–401. doi: 10.1111/pcmr.12590 28378380

[5] Moussian B. Recent advances in understanding mechanisms of insect cuticle differentiation. Insect Biochem Mol Biol. 2010;40(5):363–375. doi: 10.1016/j.ibmb.2010.03.003 20347980

[6] Wittkopp PJ, Carroll SB, Kopp A. Evolution in black and white: genetic control of pigment patterns in *Drosophila*. Trends Genet. 2003;19(9):495–504. doi: 10.1016/S0168-9525(03)00194-X 12957543

[7] Lee RC, Feinbaum RL, Ambros V. The *C. elegans* heterochronic gene lin-4 encodes small RNAs with antisense complementarity to lin-14. Cell. 1993;75(5):843–854. doi: 10.1016/0092-8674(93)90529-Y 8252621

[8] Stark A, Brennecke J, Russell RB, et al. Identification of *drosophila* microRNA targets. PLOS Biol. 2003;1(3):E60. doi: 10.1371/journal.pbio.0000060 14691535 PMC270017

[9] Leaman D, Chen PY, Fak J, et al. Antisense-mediated depletion reveals essential and specific functions of microRNAs in *Drosophila* development. Cell. 2005;121(7):1097–1108. doi: 10.1016/j.cell.2005.04.016 15989958

[10] Lai EC, Tam B, Rubin GM. Pervasive regulation of *Drosophila* Notch target genes by GY-box-, Brd-box-, and K-box-class microRNAs. Genes Dev. 2005;19(9):1067–1080. doi: 10.1101/gad.1291905 15833912 PMC1091741

[11] Jin P, Zarnescu DC, Ceman S, et al. Biochemical and genetic interaction between the fragile X mental retardation protein and the microRNA pathway. Nat Neurosci. 2004;7(2):113–117. doi: 10.1038/nn1174 14703574

[12] Nakahara K, Kim K, Sciulli C, et al. Targets of microRNA regulation in the *Drosophila* oocyte proteome. Proc Natl Acad Sci USA. 2005;102(34):12023–12028. doi: 10.1073/pnas.0500053102 16099838 PMC1189302

[13] Liu Z, Xu J, Ling L, et al. MiR-34 regulates larval growth and wing morphogenesis by directly modulating ecdysone signalling and cuticle protein in *Bombyx mori*. RNA Biol. 2020;17(9):1342–1351. doi: 10.1080/15476286.2020.1767953 32401141 PMC7549633

[14] Sugumaran M, Barek H. Critical analysis of the melanogenic pathway in insects and higher animals. Int J Mol Sci. 2016;17(10):1753. doi: 10.3390/ijms17101753 27775611 PMC5085778

[15] Futahashi R, Sato J, Meng Y, et al. Yellow and ebony are the responsible genes for the larval color mutants of the silkworm *Bombyx mori*. Genetics. 2008;180(4):1995–2005. doi: 10.1534/genetics.108.096388 18854583 PMC2600937

[16] Shirai Y, Ohde T, Daimon T. Functional conservation and diversification of yellow-y in lepidopteran insects. Insect Biochem Mol Biol. 2021;128:103515. doi: 10.1016/j.ibmb.2020.103515 33387638

[17] Lemaitre B, Hoffmann J. The host defense of *drosophila melanogaster*. Annu Rev Immunol. 2007;25(1):697–743. doi: 10.1146/annurev.immunol.25.022106.141615 17201680

[18] Nakhleh J, El Moussawi L, Osta MA. The melanization response in insect immunity. Adv Insect Physiol. 2017;52:83–109.

[19] Tanaka H, Ishibashi J, Fujita K, et al. A genome-wide analysis of genes and gene families involved in innate immunity of *Bombyx mori*. Insect Biochem Mol Biol. 2008;38(12):1087–1110. doi: 10.1016/j.ibmb.2008.09.001 18835443

[20] Lu TX, Rothenberg ME. MicroRNA. J Allergy Clin Immunol. 2018;141(4):1202–1207. doi: 10.1016/j.jaci.2017.08.034 29074454 PMC5889965

[21] Hiragaki S, Suzuki T, Mohamed AA, et al. Structures and functions of insect *arylalkylamine N-acetyltransferase* (*iaaNAT*); a key enzyme for physiological and behavioral switch in arthropods. Front Physiol. 2015;6:113. doi: 10.3389/fphys.2015.00113 25918505 PMC4394704

[22] Dai FY, Qiao L, Tong XL, et al. Mutations of an *arylalkylamine-N-acetyltransferase*, *Bm-iAANAT*, are responsible for silkworm melanism mutant. J Biol Chem. 2010;285(25):19553–19560. doi: 10.1074/jbc.M109.096743 20332088 PMC2885234

[23] Zhan S, Guo Q, Li M, et al. Disruption of an N-acetyltransferase gene in the silkworm reveals a novel role in pigmentation. Development. 2010;137(23):4083–4090. doi: 10.1242/dev.053678 21062865

[24] Long D, Lu W, Zhang Y, et al. New insight into the mechanism underlying fibroin secretion in silkworm, *Bombyx mori*. FEBS J. 2015;282(1):89–101. doi: 10.1111/febs.13105 25302556

[25] Zhao P, Dong Z, Duan J, et al. Genome-wide identification and immune response analysis of serine protease inhibitor genes in the silkworm, *Bombyx mori*. PLOS ONE. 2012;7(2):e31168. doi: 10.1371/journal.pone.0031168 22348050 PMC3278429

[26] Zou Z, Picheng Z, Weng H, et al. A comparative analysis of serpin genes in the silkworm genome. Genomics. 2009;93(4):367–375. doi: 10.1016/j.ygeno.2008.12.010 19150649 PMC2772820

[27] Guo PC, Dong Z, Xiao L, et al. Silk gland-specific proteinase inhibitor serpin16 from the *Bombyx mori* shows cysteine proteinase inhibitory activity. Biochem Biophys Res Commun. 2015;457(1):31–36. doi: 10.1016/j.bbrc.2014.12.056 25529451

[28] Pan Y, Xia H, Lu P, et al. Molecular cloning, expression and characterization of *Bmserpin-2* gene from *Bombyx mori*. Acta Biochim Pol. 2009;56(4):671–677. doi: 10.18388/abp.2009_2501 19997651

[29] Liu D, Wang L, Yang L, et al. Serpin-15 from *Bombyx mori* inhibits prophenoloxidase activation and expression of antimicrobial peptides. Dev Comp Immunol. 2015;51(1):22–28. doi: 10.1016/j.dci.2015.02.013 25720980

[30] Wang XL, Wang KL, He YY, et al. The functions of serpin-3, a negative-regulator involved in prophenoloxidase activation and antimicrobial peptides expression of Chinese oak silkworm *Dev Comp Immunol*. Dev Comp Immunol. 2017;69:1–11. doi: 10.1016/j.dci.2016.11.022 27919647

[31] Hida T, Kamiya T, Kawakami A, et al. Elucidation of melanogenesis cascade for identifying pathophysiology and therapeutic approach of pigmentary disorders and melanoma. Int J Mol Sci. 2020;21(17):6129. doi: 10.3390/ijms21176129 PMC7503925 32854423

[32] Serre C, Busuttil V, Botto JM. Intrinsic and extrinsic regulation of human skin melanogenesis and pigmentation. Int J Cosmet Sci. 2018;40(4):328–347. doi: 10.1111/ics.12466 29752874

[33] Li B, Dewey CN. RSEM: accurate transcript quantification from RNA-Seq data with or without a reference genome. BMC Bioinf. 2011;12(1):323. doi: 10.1186/1471-2105-12-323 PMC3163565 21816040

[34] Liu Z, Ling L, Xu J, et al. MicroRNA-14 regulates larval development time in *Bombyx mori*. Insect Biochem Mol Biol. 2018;93:57–65. doi: 10.1016/j.ibmb.2017.12.009 29288754

