# Supplementary material for: miR-34 regulates cuticle pigmentation by targeting Bm-iAANAT and Bmserpin3 in Bombyx mori
Source: RNA Biol. 2026 May 26;23(1):1–12. doi: 10.1080/15476286.2026.2675852 (PMC13215317; doi:10.1080/15476286.2026.2675852)
Supplement: Supplemental Material [file KRNB_A_2675852_SM8414.zip › Supplemental Table 1.docx]

**Supplement Table1. Primers used in this study.**

| **Primers name** | **Primer sequence (5' to 3')** | **Purpose** |
| --- | --- | --- |
| BmTH-F | GCTTGCCTTCCGCGTATTCCAATCGACAC | q-PCR |
| BmTH-F | AATCCGAAGCGCCGAGGGATGCAAGGCCA | q-PCR |
| BmDDC-F | ACTCGCCTCGGTTCCACGCCTACTTCCC | q-PCR |
| BmDDC-R | CTGGCAGGCCAAGCATTTGACCCAACCA | q-PCR |
| BmLac2-F | GTGTACCGTTTGTCACGCAATGTCCTAT | q-PCR |
| BmLac2-R | TGACGAACGACAATGCTGCCGTATA | q-PCR |
| BmAANAT-F | CTCGGAGACGAACTGCCGGCGCAG | q-PCR |
| BmAANAT-R | TTGACGGCGAGATTCATTGGCTCATCGC | q-PCR |
| BmPPO1-F | AGGGCCGCGATCGAGGAGGGCTACTTCC | q-PCR |
| BmPPO1-R | GCAGGAACCGGTCCCTCCAGGTCT | q-PCR |
| BmEbony-F | GGATGCGGAGACACTGGCTCCACTTACA | q-PCR |
| BmEbony-R | CATATCTCGCAGACGGAATCCACGAAAG | q-PCR |
| Bmtan-F | TTATGCTACGCTGGTCACATGCCCGGGT | q-PCR |
| Bmtan-R | GACAGAAGCTTTCTGGTTATAAATGTGCG | q-PCR |
| Bmyellow-b-F | ATGCGCTACACGCTGACATCGCTCTCG | q-PCR |
| Bmyellow-b-R | TGGAGACGTCCATTCGAAATCGATTGC | q-PCR |
| Bmyellow-d-F | CGATCGCTCATGGAGGGTGACCAACAA | q-PCR |
| Bmyellow-d-R | CTGTTGTAGATGCTAGAGAATGGAAGTAGA | q-PCR |
| Bmyellow-e-F | GATGCAACAGGCTCTGGGTACTGGATG | q-PCR |
| Bmyellow-e-R | GCACCAAATTGGTTAGCAGAGAGCTGGG | q-PCR |
| Bmyellow-fa-F | GAAGATTCTTCGTTCAATATAACAATGTGC | q-PCR |
| Bmyellow-fa-R | GGGTAAGGTCTCAAAGCCGGCGACCGTGT | q-PCR |
| Bmyellow-fb-F | TCCAAATCCAGAGGCGGTCTCGTCTCTGAC | q-PCR |
| Bmyellow-fb-R | TCCAGCTTATGTGTCAGCCCATTGTA | q-PCR |
| Bmyellow-y-F | TCTGATACCAGAGAACGCACTGCCCGTTG | q-PCR |
| Bmyellow-y-R | AATTTCGGGGATGGTTCATATGGAGC | q-PCR |
| BmDefensin-F: | GAGACGCAACCGTCTTTGACAACCA | q-PCR |
| BmDefensin-R: | ATAAGAATAGCCTTTGGGAAGACAATG | q-PCR |
| BmCecA-F: | ATGAATTTCGTACGTATTTTGAGCTTCG | q-PCR |
| BmCecA-R: | TGCCCTATGACGGCTATAGCTGGACCC | q-PCR |
| Bmcact-F: | GGTGAAAGTGGAGTGAAATCCATTAC | q-PCR |
| Bmcact-R: | GACTTTTCACAGCCGTGTACGGAGGCG | q-PCR |
| BmSpz-F: | AACGCAATACGAAGATTTGTGAGAGA | q-PCR |
| BmSpz-R: | TAGCCTTGACGATGTCTTCGGCGAAG | q-PCR |
| BmRel-F: | GAACTAAGAGCCCTTCTCTTGGAAGC | q-PCR |
| BmRel-R: | TAGTGCCGTGCTGCCACTTGTTTC | q-PCR |
| BmIMD-F: | CTGTTATGAAAGCTACCATAAAGCCGG | q-PCR |
| BmIMD-R: | CATAAATTTTATTGTCGTGTTCAAATGT | q-PCR |

| **Primers name** | **Primer sequence (5' to 3')** | **Purpose** |
| --- | --- | --- |
| Bmfadd-Q-F: | AAGTCGCACGAAGACTACAAGATAAT | q-PCR |
| Bmfadd-Q-R: | GACTTAAATTGCCACCAAATGGATTCA | q-PCR |
| BmStat-Q-F: | TCATTTCCCGATTGAAGTGAGGCATTG | q-PCR |
| BmStat-Q-R: | CTTCATCTTCGTAACGAACATGTCCGG | q-PCR |
| BmHop-Q-F: | AAAGAAAGTAATTTAGAAGCAGAGCG | q-PCR |
| BmHop-Q-R: | GTAATAGTTGCTGCCACTCTCTCATCTT | q-PCR |
| BmPi3k60-Q-F: | TGATATTGTAATTATGGCCGAGACCATG | q-PCR |
| BmPi3k60-Q-R: | TCCGAGCATAGCCCTCTCCATTGCTCT | q-PCR |
| Bmcecropin -F: | ATGAATTTCGTACGTATTTTGAGCTTCGTC | q-PCR |
| Bmcecropin -R: | CTATTTTCCTAAGGATTTCGCTTGCCCTAT | q-PCR |
| BmGloverin -F： | ATACCCCATCAGAGGCCTATTTTCAAAGCG | q-PCR |
| BmGloverin -R： | CGCGGTCATCATTGAAAATCTCTCTGTTGT | q-PCR |
| BmDefen2-F： | ATGAAGGGGGTTTACTTAATTTTCACCCTG | q-PCR |
| BmDefen2-R： | TTATTTGTCAATCTGCATCCAATTACAGGA | q-PCR |
| Bmantitrypsin -F: | ACTCCCTCAGCTCAGCCACAGCGGCTGTTC | q-PCR |
| Bmantitrypsin -R: | CTCTCCATATTTATAATCGCCGCGTTTATA | q-PCR |
