# Supplementary figures and images for: miR-34 regulates cuticle pigmentation by targeting Bm-iAANAT and Bmserpin3 in Bombyx mori
Source: RNA Biol. 2026 May 26;23(1):1–12. doi: 10.1080/15476286.2026.2675852 (PMC13215317; doi:10.1080/15476286.2026.2675852)

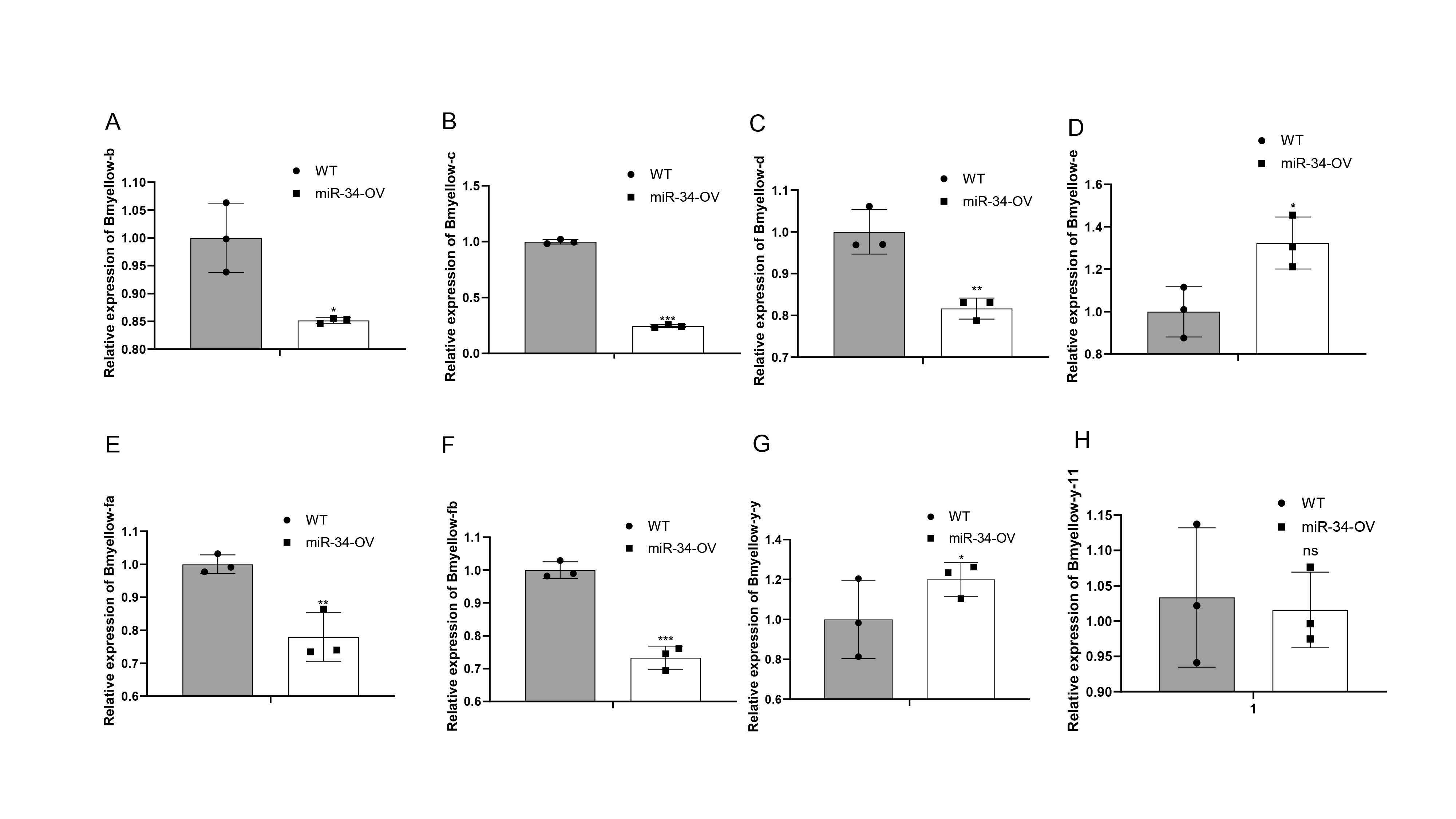

Supplement: Supplemental Material [file KRNB_A_2675852_SM8414.zip › Supplement Fig2.tif]

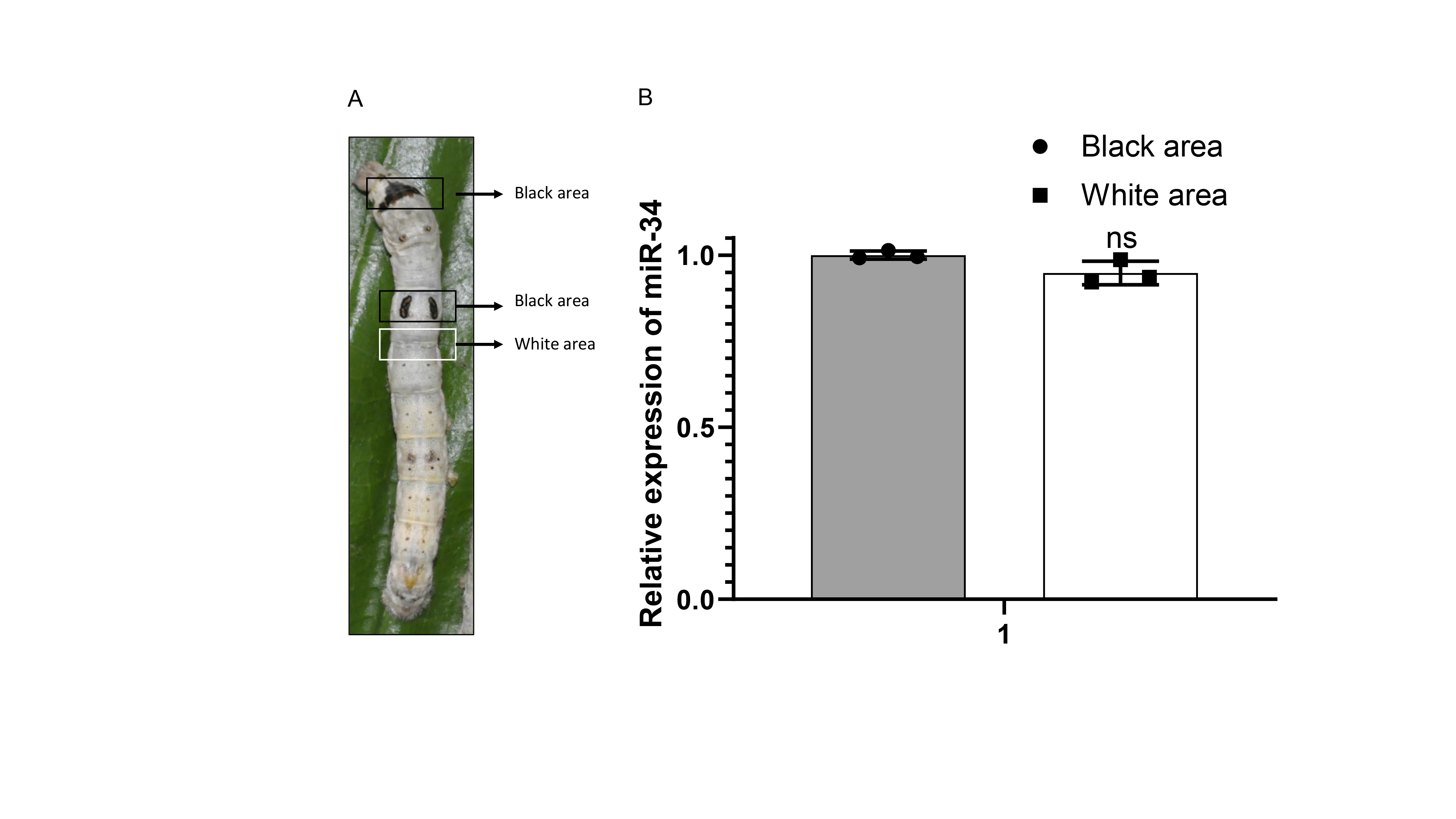

Supplement: Supplemental Material [file KRNB_A_2675852_SM8414.zip › Supplement Fig1.tif]
